# Human Dental Pulp Cells Differentiate toward Neuronal Cells and Promote Neuroregeneration in Adult Organotypic Hippocampal Slices In Vitro

**DOI:** 10.3390/ijms18081745

**Published:** 2017-08-11

**Authors:** Li Xiao, Ryoji Ide, Chikako Saiki, Yasuo Kumazawa, Hisashi Okamura

**Affiliations:** 1 The Nippon Dental University, School of Life Dentistry at Tokyo, 1-9-20 Fujimi, Chiyoda-ku, Tokyo 102-0071, Japan; ryo-ide@tky.ndu.ac.jp (R.I.); chikako@tky.ndu.ac.jp (C.S.); 2 Department of Oral and Maxillofacial Surgery, The Nippon Dental University Hospital, 2-3-16 Fujimi, Chiyoda-ku, Tokyo 102-8159, Japan; yasuo-kumazawa@n05.itcom.net (Y.K.); hisashi124@gmail.com (H.O.)

**Keywords:** human dental pulp cells, adult hippocampal slice culture, neuronal regeneration, co-cultivation

## Abstract

The adult mammalian central nerve system has fundamental difficulties regarding effective neuroregeneration. The aim of this study is to investigate whether human dental pulp cells (DPCs) can promote neuroregeneration by (i) being differentiated toward neuronal cells and/or (ii) stimulating local neurogenesis in the adult hippocampus. Using immunostaining, we demonstrated that adult human dental pulp contains multipotent DPCs, including STRO-1, CD146 and P75-positive stem cells. DPC-formed spheroids were able to differentiate into neuronal, vascular, osteogenic and cartilaginous lineages under osteogenic induction. However, under neuronal inductive conditions, cells in the DPC-formed spheroids differentiated toward neuronal rather than other lineages. Electrophysiological study showed that these cells consistently exhibit the capacity to produce action potentials, suggesting that they have a functional feature in neuronal cells. We further co-cultivated DPCs with adult mouse hippocampal slices on matrigel in vitro. Immunostaining and presto blue assay showed that DPCs were able to stimulate the growth of neuronal cells (especially neurons) in both the CA1 zone and the edges of the hippocampal slices. Brain-derived neurotrophic factor (BDNF), was expressed in co-cultivated DPCs. In conclusion, our data demonstrated that DPCs are well-suited to differentiate into the neuronal lineage. They are able to stimulate neurogenesis in the adult mouse hippocampus through neurotrophic support in vitro.

## 1. Introduction

The adult mammalian central nerve system (CNS) presents inherent difficulties for its effective regeneration following traumatic injuries or neurodegenerative diseases, such as spinal cord injury, stroke and Alzheimer’s disease [1]. The greatest problem in overcoming neuronal damage and achieving successful CNS recovery is the difficulty in regenerating functional neurons in the CNS [2,3,4]. Exogenous cellular replacement and endogenous cell stimulation were considered the fundamental approaches for neuroregeneration. Stem cell-based therapies were pursued by these two approaches to achieve neuroregeneration: (i) replacing and/or promoting the survival of damaged cells; (ii) providing trophic support to stimulate local neurogenesis [5,6,7].

The transplantation of human embryonic stem (ES) cells, neural stem and progenitor cells (NSPCs), oligodendrocyte precursor cells (OPCs), and bone marrow-derived mesenchymal stem cells (BMSCs) can improve recovery outcomes in animal models of spinal cord injury, and may enhance neurogenesis or replace lost neurons in neurodegenerative diseases [8,9,10,11,12]. However, little is known about the interaction between transplanted stem cells and local neuronal tissues. To date, there is no direct evidence demonstrating that stem cell-based therapies could improve neurogenesis in neuronal tissues, either in vivo or in vitro.

Human adult dental tissue is derived from the neural crest, a distinctive transient embryonic cell population. During embryonic development, the neural crest cells give rise to a prodigious number of differentiated cell types including neuronal cells, osteoblasts and dental mesenchymal cells [13,14,15]. It has been reported that the dental mesenchymal cells generate approximately 90% of the dental pulp cells [16]. Thus, dental pulp cells and neuronal cells share the same origin. Dental pulp cells (DPCs) are commonly obtained from extracted third molars and primary teeth, which are usually discarded as medical waste. Compared to other stem cells (such as NSPCs and BMSCs), DPCs are much easier to obtain. Numerous studies demonstrated that DPCs exhibit multipotency, including osteogenic, odontogenic, neurogenic and pancreatic potency [17,18,19,20,21], and have been successfully used for bone tissue engineering [22,23]. DPCs can be easily cryopreserved and stored for long periods of time without losing their multipotency [24]. We and others have demonstrated that DPCs express neural crest stem cell marker P75 and neuronal marker HuC/D, even without neuronal induction [25,26]. It has been reported that DPCs can exhibit better neural stem cell properties in comparison to BMSCs. [27]. In vitro studies have demonstrated the capability of monolayer DPCs to differentiate into neuronal cells with the expression of neuronal markers and electrophysiologial features [14,20]. These evidences suggest that DPCs are a suitable cell source for neuronal regeneration.

In the present study, we examined the neuronal differentiation potential of DPCs under neuronal inductive conditions in a three-dimensional environment. We also co-cultivated DPCs with adult mouse hippocampal slices in vitro to observe the possible interaction between DPCs and hippocampal neurons by our innovative method using the hippocampal slice culture. This study demonstrated that DPCs have an ability to differentiate toward neuronal lineage and can stimulate neurogenesis in the adult mouse hippocampus by providing neurotrophic support.

## 2. Results

### 2.1. Distribution of Stem Cells in Adult Human Dental Pulp

**Figure 1:**
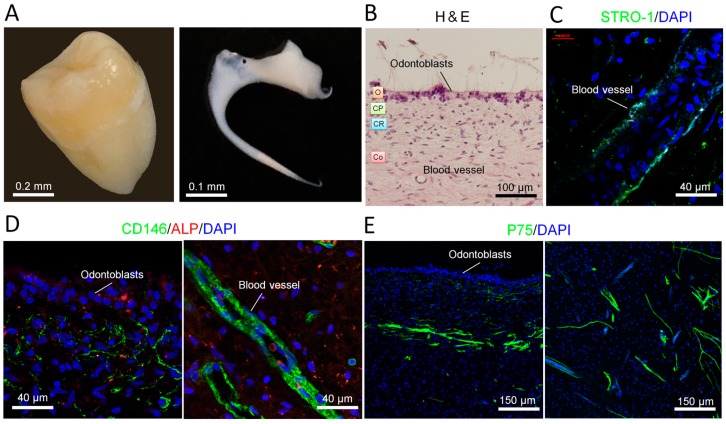
Characterization of human dental pulp: stem cell distribution. (**A**) Digital photos of a human third molar tooth (left) and a human dental pulp from third molar (right); (**B**) Human dental pulp was stained with hematoxylin and eosin (H&E). O, odontoblastic zone; CP, cell poor zone; CR, cell rich zone; Co, core of the pulp; (**C**) Mesenchymal stem cell marker STRO-1 was expressed along the blood vessel. Nuclei were stained with DAPI (4′,6-diamidino-2-phenylindole); (**D**) Stem cell marker CD146 was expressed at the places near the odontoblasts and along the blood vessel. Alkaline phosphatase (ALP, marker for skeletal mineralization) was detected in the odontogenic zone; (**E**) Neuronal crest stem cell marker P75 was expressed in the core of dental pulp.

We obtained human dental pulp from the third molar (Figure 1A). The isolated dental pulp presented typical pulp architecture: odontogenic, cell-poor and cell-rich zones and the core (Figure 1B). Immunostaining showed that mesenchymal stem cell markers STRO-1 (Figure 1C) and CD146 (also a vascular marker) (Figure 1D) were both expressed along the blood vessels. CD146 was also expressed in the cell-rich zone. Moreover, neuronal crest stem cell marker P75 was expressed in the core of the pulp (Figure 1E). These three stem cell markers were expressed in different places in the pulp, suggesting that human dental pulp contains different progenitors.

### 2.2. Parallel Multilineage Differentiation in DPC-Formed Spheroids under Osteogenic Medium

**Figure 2:**
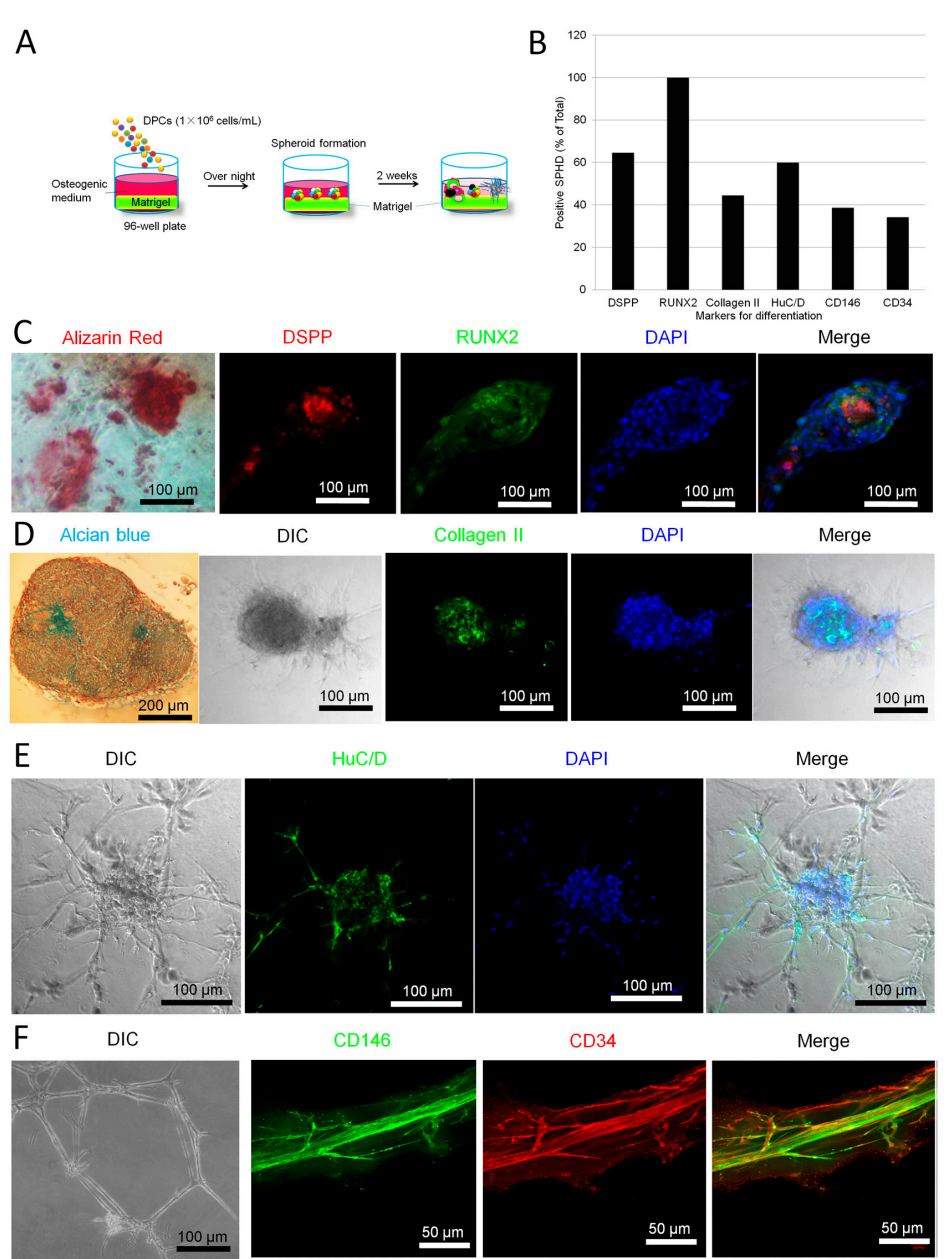
Multi-differentiation of dental pulp cell (DPC)-formed spheroids. Human dental pulp cells (1 × 10^6^) were seeded on matrigel in osteogenic medium overnight. The cells spontaneously formed spheroids. After further cultivation for 2 weeks, the spheroids differentiated into multi-lineage cell masses. (**A**) Experimental setup; (**B**) The spheroids were cultivated in osteogenic medium in 96-well plates for 14 days. The spheroids were followed by alizarin red, alcian blue and immunofluorescence staining. Five wells were prepared for each marker. The markers (antibodies) include DSPP, RUNX2, collagen II, HuC/D, CD146 and CD34. The positive cell masses for each marker were counted in every 10 spheroids per well × 5 wells/antibody; (**C**–**F**) Typical images of differentiated spheroids (*n* = 5). Data represent three independent experiments.

We isolated dental pulp cells (DPCs) from the dental pulp according to our previous report [28]. The cells showed typical fibroblastic morphology when being cultivated as monolayer. When we seeded the cells on matrigel-coated 96-well culture plates, the cells formed small sized spheroids with a diameter of 100–300 µm. We then cultivated the spheroids in osteogenic inductive medium for about 2 weeks. Figure 2 showed that, after 2 weeks osteogenic induction, the small-sized spheroids showed various shapes and expressed different markers including the osteogenic markers DSPP (dentin sialophosphoprotein) and RUNX2 (runt-related transcription factor 2) (Figure 2C), cartilaginous marker collagen II (Figure 2D), neuronal marker HuC/D (Figure 2E) and vascular markers CD146 and CD34 (Figure 2F). Some spheroids were positive to alizarin red staining (Figure 2C). Some spheroids were stained by alcian blue (Figure 2D). Some spheroids presented neuronal cell-like morphology (Figure 2E) while others formed blood vessel-like tubular structures (Figure 2F). Semi-quantitative analysis showed that the spheroids expressed various markers at different levels (Figure 2B). Our data suggest that, under osteogenic medium (which contains dexamethasone, an essential reagent for various tissues’ development), the spheroids differentiate into neuronal, vascular, osteogenic and cartilaginous cell masses.

### 2.3. Neuronal Differentiation of DPC-Formed Spheroids

**Figure 3:**
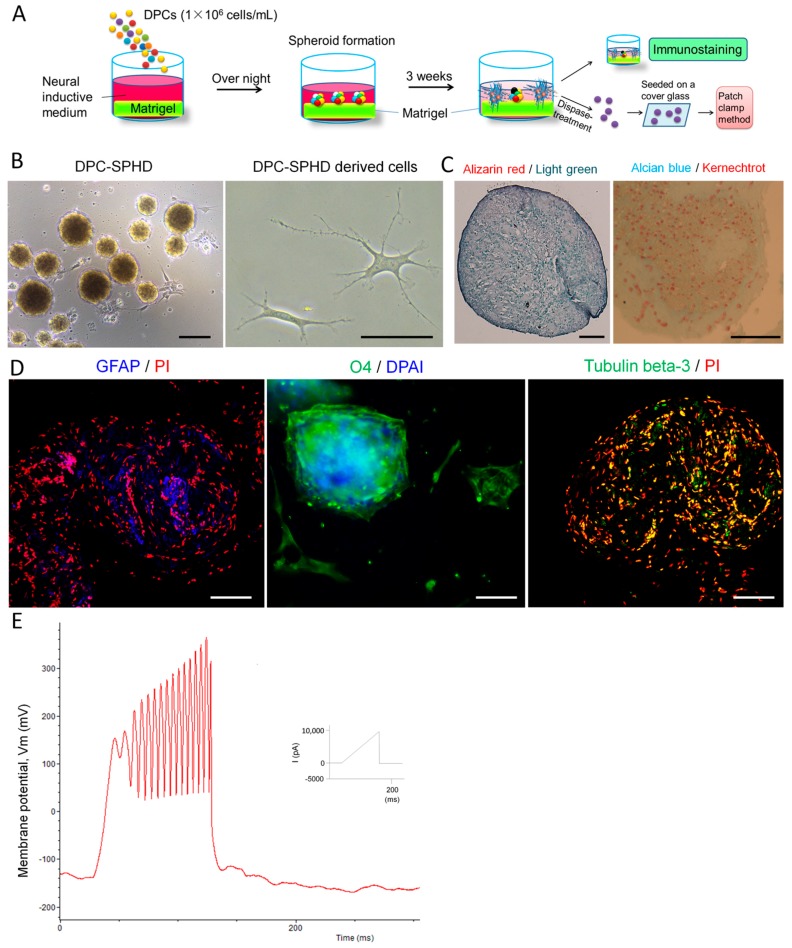
Expression of neural markers for differentiation and electrophysiological properties in DPC-formed spheroids on matrigel. DPCs were seeded on matrigel in neural inductive medium overnight. The cells spontaneously formed spheroids in 96-well culture plates and were further cultivated for 3 weeks. (**A**) Experimental setup; (**B**) The spheroids in culture for 3 days. Scale bar = 50 µm; (**C**) Three weeks after neuronal induction, the spheroids were stained with alizarin red (nuclei were stained with light green) and alcian blue (nuclei were stained with kernechtrot). The spheroids were not stained by both alizarin red and alcian blue. Scale bar = 10 µm; (**D**) Expression of neural markers for differentiation in the spheroids after 3 weeks cultivation (immunofluorescence staining). Images were taken by LSM. The spheroids expressed the glia cell marker, GFAP, oligodendrocyte, O4, and the neuron marker, tubulin β-3. Nuclei were stained with propidium iodide (PI) or DAPI. Scale bar = 25 µm. DPC-SPHD, DPC-formed spheroids; (**E**) Electrophysiological characteristics of DPC-formed spheroids. Patch-clamp recordings of spheroid-derived cells. After being cultivated in neural inductive media for 3 weeks, spheroids were decomposed into single cells by dispase-treatment. The cells were seeded on a cover glass overnight to adhere to the glass surface. Action potential properties were analyzed by whole-cell patch-clamp recordings. Membrane potential oscillations were generated by ramp protocol using the current clamp mode with 10 nA maximal amplitude in spheroid-derived cells.

We cultivated DPCs in neuronal inductive medium. DPCs can form spheroids after being seeded on matrigel-coated culture surfaces in neuronal inductive medium (Figure 3A,B). Two weeks after cultivation, the spheroid-derived cells presented neuronal cell-like morphology (Figure 3B). Immunofluorescence staining showed that DPC-formed spheroids expressed neuronal markers GFAP (glial fibrillary acidic protein), O4 (oligodendrocyte marker 4) and tubulin β-3 (Figure 3D). Electrophysiological study demonstrated that cells which are derived from the spheroids exhibited the capacity to consistently produce action potentials, suggesting these cells were functional neuronal cells (Figure 3E). The spheroids were negative to both alizarin red and alcian blue staining (Figure 3C). The vascular structures were also absent (data not shown).

### 2.4. Promotion Effect of DPCs on Neurogenesis in Organotypic Hippocampal Slices

**Figure 4:**
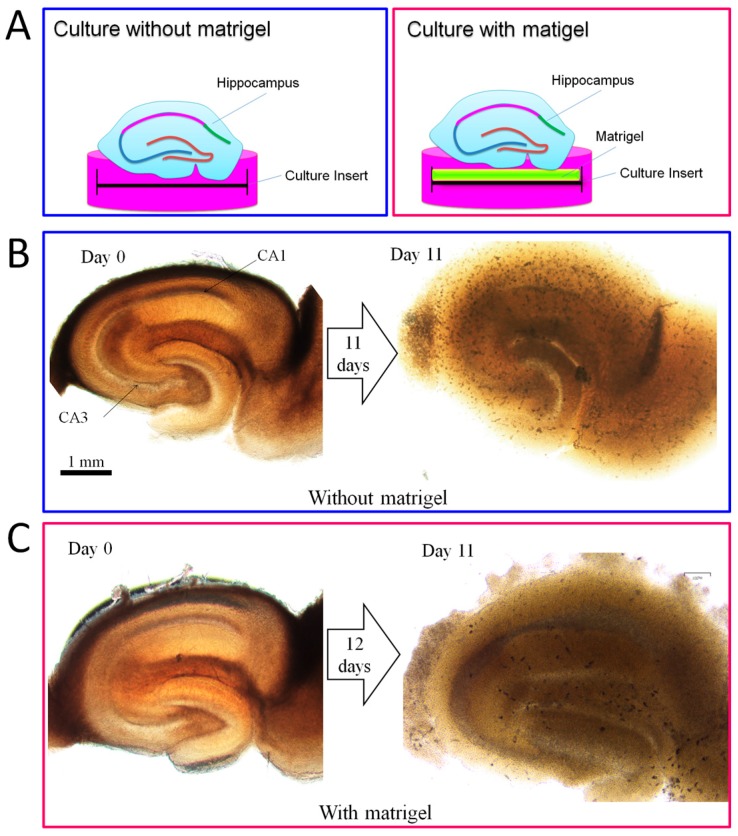
A novel method for long-term culture of adult mouse hippocampal slices in vitro. (**A**) Experimental setup; (**B**) Adult mouse hippocampal slices were cultivated on the culture insert without matrigel. After 11 days of cultivation, the CA1 and CA3 zones had disappeared; (**C**) Adult mouse hippocampal slices were cultivated on a matrigel-coated culture insert. After 11 days of cultivation, the CA1 and CA3 zones were still clear.

We invented a novel method to maintain adult mouse hippocampal slices in vitro for long-term culture by using a matrigel-coated culture insert. As shown in Figure 4B, without matrigel, adult mouse hippocampal slices lost their anatomical structure after 11 days of cultivation, whereas with matrigel the hippocampal slices could keep the anatomical properties of their corresponding hippocampal circuits (Figure 4C). We have observed that the hippocampal slices can keep a clear structure of CA1 and CA3 zones for more than one month on the matrigel-coated culture insert (data not shown).

**Figure 5:**
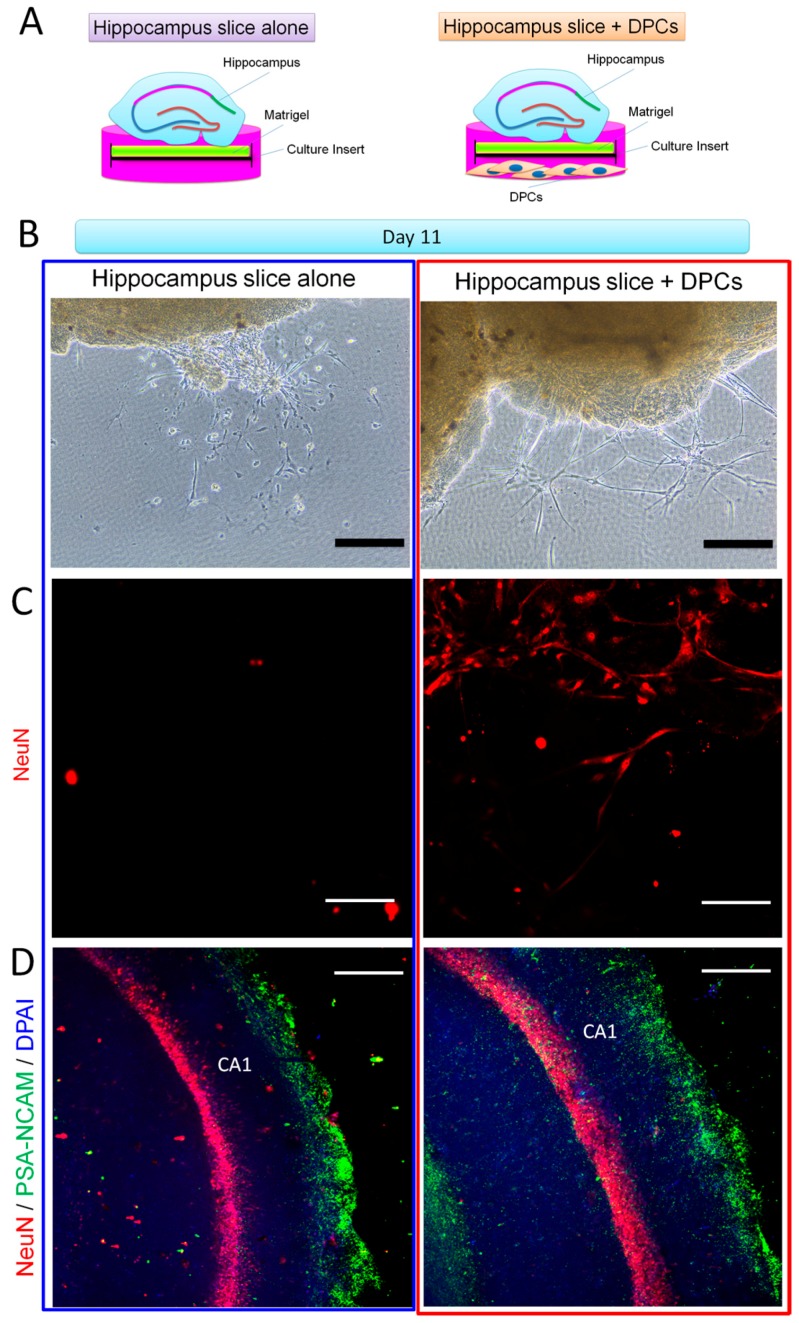
Effect of DPCs on neurogenesis in adult mouse hippocampal slices in vitro. (**A**) Experimental setup. Mouse hippocampus slices were cultivated on matrigel with DPCs or by themselves for 11 days, followed by immunofluorescence staining; (**B**) Images were taken by a phase contrast microscope. Hippocampus slice-derived cells showed glia cell-like morphology when cultivated alone (left). However, when the hippocampus slice co-cultivated with DPCs, the derived cells exhibited neuron-like morphology and formed networks. Scale bar = 50 µm (**C**) Immunofluorescence staining showed that cells derived from the co-cultivated hippocampus slice are positive to the mature neuron mark NeuN (right). However, cells derived from the single cultivated hippocampus did not react with NeuN (left). Images were taken by LSM. Scale bar = 25 µm; (**D**) In solo or co-cultivated hippocampus slices, cells reacted with NeuN and PSA-NCAM (a marker of developing and migrating neurons) antibodies. Images were taken by LSM. Scale bar = 50 µm.

**Figure 6:**
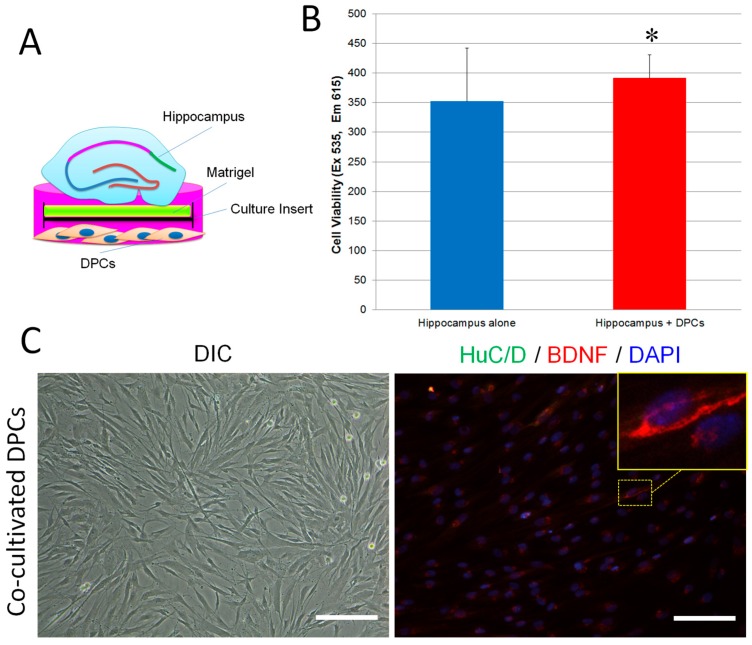
DPCs promoted cell growth in adult mouse hippocampal slices in vitro and expressed BDNF. (**A**) Experimental setup; (**B**) Adult hippocampal slices were cultivated on matrigel-coated culture inserts (5 slices/well) with or without DPCs for 17 days. The culture inserts were transferred into new 6-well plates and followed by presto blue assay. Data are presented as mean ± SD. * *p* < 0.05; (**C**) DPCs kept fibroblast morphology while co-cultivated with hippocampal slices (left, magnification = 20×). Immunofluorescence staining showed that some DPCs were positive to anti-BDNF antibodies. The expression of HuC/D was hardly detected in co-cultivated DPCs (right, magnification = 20×). Scale bar = 50 µm

We then co-cultivated DPCs and the hippocampal slices in neuronal inductive medium for 11 days and observed their cellular properties (Figure 5A). As shown in Figure 5B, both solo and co-cultivated hippocampal slices derived new cells along the edges. However, the hippocampal slice-derived cells between the two culture systems showed different morphologies: the solo-culture-derived cells presented small and short dendrites, whereas the co-culture-derived cells exhibited long dendrites and formed a network-like structure. Immunostaining showed that cells derived from the co-culture were positive for anti-NeuN antibody (a biomarker for mature neurons [29]) suggesting they were mature neurons. However, the expression of NeuN in solo-culture-derived cells was hardly detected (Figure 5C). We further observed that, compared to solo-cultivated hippocampal slices, the co-cultivated hippocampal slices had a wider CA1 zone and much more cells which were positive to both anti-NeuN and anti-PSA-NCAM (polysialylated-neural cell adhesion molecule, a marker for developing and migrating neurons) antibodies (Figure 5D). Cell viability assay showed that cells in the co-cultures had a significantly higher proliferation rate than the solo-cultures (Figure 6B). BDNF is an important molecule for the survival of neurons [30]. We observed that co-cultivated DPCs (Figure 6A) expressed BDNF with a Golgi-associated labeling pattern (Figure 6C), suggesting DPCs could secrete BDNF. These data suggest that DPCs could promote neuronal survival and stimulate cell growth in co-cultivated organotypic hippocampal slices by providing neurotrophic support.

## 3. Discussion

Human dental pulp is a gelatinous embryonic type of connective tissue that contains several types of cells, including fibroblasts, odontoblasts, mesenchymal cells and extravasated leukocytes and macrophages [31]. In the present study, we demonstrated that adult human dental pulp contains cells which express STRO-1 (the best-known mesenchymal stem cells marker) [32], CD146 (endothelial cells and mesenchymal stem cells marker) [33,34] and P75 (neural crest stem cells marker) [35]. Both STRO-1 and CD146 were expressed along the blood vessels. CD146 was also expressed in the cell-rich zone. P75 was mainly expressed in the pulp core (Figure 1). This finding suggests that the adult human dental pulp contains different types of mesenchymal stem cells/progenitors. Cells isolated from the dental pulp can automatically form spheroids on matrigel. These DPC-formed spheroids presented various morphologies and expressed neuronal, vascular, osteogenic and cartilaginous under osteogenic induction, indicating that DPCs could exhibit multi-potency under certain conditions (Figure 2). As is well known, stem cells [including embryonic stem (ES) cells, induced pluripotent stem (iPS) cells and adult stem cells) usually differentiate into a single lineage in a monolayer culture system. The spontaneous multi-differentiation of stem cells is normally observed in vivo (for example, ES cells transplantation-induced teratoma formation). Here, our data showed that, under matrigel-based culture conditions, DPC-spheroids differentiated into multi-lineage spontaneously in the osteogenic medium. This evidence suggested that matrigel-based culture conditions can offer an in vivo-like microenvironment. However, under neuronal induction, DPC-formed spheroids only differentiated into neuronal lineage and exhibited an electrophysiological characteristic of neuronal cells (Figure 3), indicating that DPCs are more suitable for neuronal differentiation.

Organotypic brain slice cultures are considered very useful tools for investigating the cellular and molecular processes of CNS in vitro [36]. In particular, hippocampal organotypic slice cultures can present a well-defined cellular architecture of the hippocampal circuit, which preserves in vivo-like situations [37]. Hippocampal organotypic slices from embryonic (>E14) and postnatal (<P12) rats or mice are commonly used for neuroscientific research. However, little is known about hippocampal slices obtained from adult animals, because the cells, especially neurons in the slices, have difficulty surviving even short-term cultivation [38]. In this study, we invented a novel method to maintain adult mouse hippocampal slices in vitro for long-term culture by using a matrigel-coated culture insert. Using the method, adult mouse hippocampal slices can keep the anatomical and cellular properties of their corresponding hippocampal circuits for more than 11 days (Figure 4). Matrigel contains a mixture of extracellular matrix (ECM) proteins which can improve graft survival, repair damaged tissues and support the growth and maintenance of a variety of cells [39,40]. Thus, we think that matrigel can keep hippocampal slices healthy for long-term cultivation due to it offers the fundamental support of ECM in vitro. Using our method, we further investigated whether DPCs provide environmental enrichment to support host neurons in the adult organotypic hippocampal slices, and we co-cultivated DPCs with the hippocampal slices. Our data showed that DPCs can stimulate the proliferation of neuronal cells (mainly neurons) both along the edge and inside the hippocampal slices (Figure 4). DPCs expressed neurotrophic factor BDNF in the Golgi complex, but not the neuronal marker HuC/D. DPCs also did not present neuronal cell-like morphology. These data suggest that, when being co-cultivated with the hippocampus, instead of differentiating toward neuronal lineage, DPCs preferred secreting neurotrophic factors to provide in situ support (Figure 5).

In conclusion, our results suggest that DPCs are multi-potent cells. They are well-suited to differentiate into neuronal cells. DPCs are able to stimulate neurogenesis in the hippocampus of adult mice by providing neurotrophic support. Our method for the long-term culture of adult hippocampal slices is an innovative and important tool for studying neurogenesis, neuroprotection and neurodegeneration in vitro. This work allows for functional studies to explore the cellular and molecular basis for stem cell-based therapies related to neuronal cell death, neuroprotection, and synaptic plasticity.

## 4. Materials and Methods

### 4.1. Cell Culture

To obtain human dental pulp cells, lower third molars were obtained from adults (17–26 years old) at the Nippon Dental University Hospital at Tokyo under approved guidelines set by the Committee of Ethics, the Nippon Dental University School of Life Dentistry at Tokyo (authorization number: NDU-T2012-35, 13 August 2015). Dental pulp tissues (Figure 1b) were minced into 1- to 3-mm^2^ fragments, plated on 10-cm dishes with the complete growth medium (MEM-α (Thermo Fisher Scientific, Tokyo, Japan) containing 20% FBS, 100 units/mL penicillin, 10 mg/mL streptomycin and 1% Gibco^®^ GlutaMAX™ Supplement (Thermo Fisher Scientific)), and cultured at 37 °C in a humidified tissue culture incubator with 5% CO_2_ and 95% O_2_. After 7–10 days, the plastic-adherent confluent cells were treated with 0.05% trypsin containing 1 mM EDTA for 5 min to harvest pure mesenchymal cells. The dental pulp cells (DPCs) were passaged and continuously subcultured and maintained in the complete growth medium. DPCs from third to seventh passages were used in the experiments.

### 4.2. 3D Culture of Spheroids on Matrigel

200 µL matrigel (354234, Corning) was added into a 24-well insert (BD Bioscience, Tokyo, Japan) which was placed in the culture well and set at 37 °C for 30 min. DPCs (1 × 10^6^ cells/mL) were inoculated on the gels and cultivated in osteogenic medium (MEM-α containing 10% FBS, 50 µM ascorbic acid 2-phosphate, 0.1 µM dexamethasone and 10 mM β-glycerophosphate) or neural inductive medium (Neurobasal^®^ Medium (Thermo Fisher Scientific) containing B-27 Supplement (Thermo Fisher Scientific)) [41]. Cultures were fed every 2–3 days by replacing the medium.

### 4.3. Organotypic Hippocampus Slice Culture

24 Jcl:ICR mice (3–4 weeks old) were purchased from CLEA Japan, Inc. (Tokyo, Japan) and used in this study. The animal experiments were approved by the Animal Ethics Committees, the Nippon Dental University School of Life Dentistry at Tokyo (authorization number: 16-02-1, 13 May 2016). To prepare the hippocampi slices, we used sharp utility scissors to cut the head of the animal and scooped out the brain quickly with a rounded spoon micro spatula and place it into the ice cold dissecting solution (Containing 1 mM CaCl_2_, 10 mM D-Glucose, 4 mM KCl, 5 mM MgCl_2_, 26 mM NaHCO_3_, 234 mM sucrose and 0.1% *v*/*v* phenol red solution 0.5% in DPBS) [42]. We then separated the hippocampi from the brain and cut the hippocampi to slices with a thickness of 250 µM with a McIlwain Tissue Chopper (The Mickle Laboratory Engineering Co. LTD, Guildford, UK). The hippocampi slices were then gently transferred onto six-well culture inserts (PICM0RG50, Millipore, Tokyo, Japan) which were coated with 200 µL matrigel (354234, Corning, Tokyo Japan). The hippocampi slices were cultivated in 900 µL neural medium (Neurobasal^®^ Medium (Thermo Fisher Scientific) containing B-27 Supplement (Thermo Fisher Scientific)) at 35 °C in a humidified tissue culture incubator with 5% CO_2_ and 95% O_2_. For co-cultivation, DPCs (4 × 10^4^ cells/well) were seeded into a six-well plate two days before the hippocampi slices culture and then co-cultivated with hippocampi slices as shown in Figure 4A.

### 4.4. Immunohistochemistry

Immunohistochemistry staining was performed with Histostain™ kit (Thermo Fisher Scientific, Inc.) according to the manufacturer’s protocol. Briefly, specimens were deparaffinized in xylene and dehydrated in a graded series of ethanol. The endogenous peroxidase activity was quenched by using 3% hydrogen peroxide in methanol. Specimens were incubated with serum blocking solution for 10 min to suppress the non-specific binding of IgG, and then incubated for 60 min with saturating levels of primary antibodies. The primary antibodies used were anti-STRO-1 (R&D Systems, MAB1038, Minneapolis, MN, USA), anti-CD146 (Novocastra, Newcastle, UK), anti-ALP (Abcam, ab65834, Tokyo, Japan), anti-P75 (Abcam, ab8877), anti-DSPP (Sigma-Aldrich, HPA036230, Tokyo Japan), anti-Runx2 (Abcam, ab23981), anti-collagen II (Millipore, MAB8887), anti-HuC/D (Thermo Fisher Scientific, A-21271), anti-CD34 (Millipore, CBL496), anti-tubulin β-3, anti-GFAP and anti-O4 (R&D systems, SC028), anti-NeuN (Abcam, ab177487), anti-PSA-NCAM (Thermo Fisher Scientific, 14-9118-80) and anti-BDNF (Abcam, ab108319). For immunofluorescence staining, specimens were reacted with fluorochrome-conjugated secondary antibody (Thermo Fisher Scientific, A11001, A11012, A-21097, A-21469) diluted to 2 µg/mL in PBS with 1.5% normal blocking serum. The nuclei were stained with DAPI.

Whole mount fluorescence immunohistochemical staining was performed using a standard protocol [43]. Samples were imaged and analyzed with a confocal laser scanning microscopy (LSM700, Carl Zeiss Microscopy Co., Ltd., Tokyo, Japan).

### 4.5. Histological Analysis

Samples were fixed in 10% (*v*/*v*) buffered formalin for histological analyses. The specimens were embedded in paraffin, cut into about 5mm thick sections, then followed with hematoxylin and eosin (H&E) staining, and alician blue and alizarin red S staining for morphological analysis. Images were taken by a bio-imaging navigator (Olympus, FSX100, Tokyo, Japan).

### 4.6. Whole-Cell Patch Recording

At the completion of the neural differentiation assay, cells were liberated with dispase (spheroids) or typsin (monolayer cells) and seeded onto glass coverslips treated with hydrochloric acid at a concentration of 5 × 10^4^ cells/ mL in neural inductive medium or regular medium and incubated overnight. Whole-cell patch clump recordings were performed using an Axopatch 200B amplifier (Molecular Devices, Tokyo, Japan) at room temperature (21–23 °C). Data were sampled at 20 kHz and digitized with a Digidata 1440A interface and recorded using pCLAMP10.2 software (Molecular Devices). Data were low-pass filtered at 5 kHz. Patch pipettes were pulled from borosilicate glass capillaries on a NARISHIGE PC-10 puller and had resistances of 2–4 MΩ. Series resistance was compensated by at least 80%. For recoding action potentials, ramp protocol was applied by using current clamp mode with 10 nA maximal amplitude. The bath solution contained (in mM): NaCl, 160; KCl, 5; CaCl_2_, 2; MgCl_2_, 1; glucose, 10; and HEPES, 10; adjusted to pH 7.4 with NaOH.

### 4.7. Cell Viability Assay

Cell viability in cultivated hippocampal slices were measured by presto blue assay according to the standard protocol. Hippocampal slices were cultivated on six-well culture inserts (five slices/insert) with or without DPCs for 3 weeks. The culture inserts were then moved to a new six-well plate and incubated for 3 h with fresh culture medium (900 µL) supplemented with 10 vol.% PrestoBlue^®^ (Thermo Fisher Scientific, A13261). The PrestoBlue^®^ reduction by the cells in the hippocampal slices expressed as fluorescence intensity units was measured on a microplate reader (SH-9000Lab, Hitachi, Tokyo, Japan) with excitation 560 nm and emission 590 nm.

### 4.8. Statistical Analysis

All data, expressed as mean ± SD, were processed statistically by GNU PSPP statistical analysis Software (version 0.8.2-gad9374) and the OpenStat program by Bill Miller. A one-way analysis of variance followed by least significant difference test (equal variances assumed) or the Dunnett’s T3 test (equal variances not assumed) was used for statistical analysis. The differences of the data were considered when *p* < 0.05.
